# The impact of delayed gastric emptying as measured by gastric emptying scintigraphy on the outcome of magnetic sphincter augmentation

**DOI:** 10.1007/s00464-023-10190-y

**Published:** 2023-06-16

**Authors:** Sven E. Eriksson, Ping Zheng, Inanc S. Sarici, Xinxin Shen, Blair A. Jobe, Shahin Ayazi

**Affiliations:** 1 Esophageal Institute, Allegheny Health Network, 4815 Liberty Avenue, Suite 439, Pittsburgh, PA 15224 USA; 2 Department of Surgery, Drexel University, Philadelphia, PA USA

**Keywords:** Gastroparesis, Delated gastric emptying, Magnetic sphincter augmentation, Outcome, Bloating

## Abstract

**Introduction:**

The impact of delayed gastric emptying (DGE) on the outcome of anti-reflux surgery (ARS) is controversial. There is concern that poor gastric emptying diminishes outcomes. Magnetic sphincter augmentation (MSA) may have a comparatively mild impact on gastric physiology, but the relationship between DGE and MSA outcomes is unknown. This study aims to evaluate the relationship between objective DGE and MSA outcomes over time.

**Methods:**

Patients who completed gastric emptying scintigraphy (GES) prior to MSA between 2013 and 2021 were included. DGE was defined as a 4 h retention > 10% or half emptying time > 90 min on GES. Outcomes were compared between DGE and normal gastric emptying (NGE) groups at 6 months, 1 and 2 years. Sub-analysis of patients with severe (> 35%) DGE and correlation analysis between 4-h retention and symptom and acid-normalization were performed.

**Results:**

The study population consisted of 26 (19.8%) patients with DGE and 105 with NGE. DGE was associated with more 90-days readmissions (18.5 vs 2.9%, *p* = 0.009).

At 6 months patients with DGE had higher median (IQR) GERD-HRQL total [17.0(10–29) vs 5.5(3–16), *p* = 0.0013], heartburn [1(1–3) vs 0(0–1), *p* = 0.0010) and gas-bloat [4(2–5) vs 2(1–3), *p* = 0.033] scores.

Outcomes at 1 and 2 years follow-up were comparable (*p* > 0.05). From 6 months to 1-year the gas-bloat score decreased from 4(2–5) to 3(1–3), *p* = 0.041. Total and heartburn scores decreased, but not significantly.

Severe DGE (*n* = 4) patients had lower antiacid medication freedom at 6 months (75 vs 87%, *p* = 0.014) and 1-year (50 vs 92%, *p* = 0.046). There were non-significant trends for higher GERD-HRQL scores, dissatisfaction, and removal rates in severe DGE at 6 months and 1-year.

There was a weak correlation between 4-h retention and 6-month GERD-HRQL total score [*R* = 0.253, 95%CI (0.09–0.41), *p* = 0.039], but not acid-normalization (*p* > 0.05).

**Conclusion:**

Outcomes after MSA are diminished early on in patients with mild-to-moderate DGE, but comparable by 1 year and durable at 2 years. Severe DGE outcomes may be suboptimal.

**Graphical abstract:**

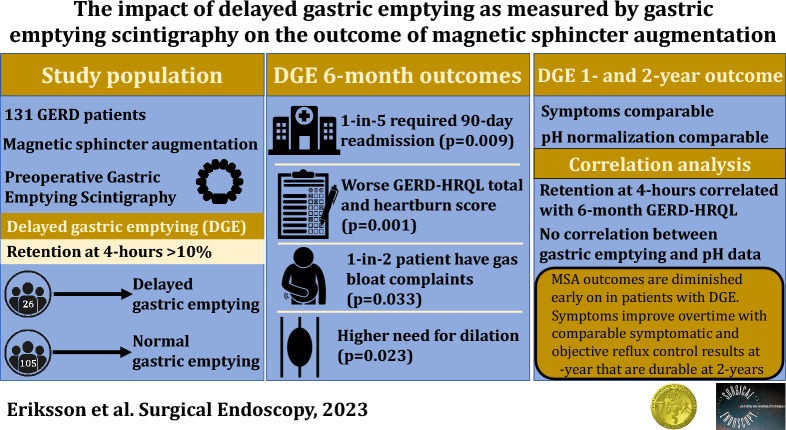

Delayed gastric emptying (DGE) is a defining characteristic of gastroparesis and a common finding in patients with gastroesophageal reflux disease (GERD). In fact, one-in-ten patients referred for antireflux surgery are found to have DGE on gastric emptying scintigraphy (GES) [1, 2]. However, the impact of objective DGE on ARS is controversial. Several studies have demonstrated higher recurrence rates among patients with DGE after fundoplication [2–4]. However, some studies have demonstrated no detrimental impact on ARS outcomes [5].

Given this controversy and the unclear implications of DGE surgeons often adopt a cautious approach during the preoperative workup of a patient with suspected DGE. A major fear is that patients with DGE may be prone to develop gas-bloat syndrome and exacerbation of gastroparesis symptoms after surgery. Antireflux surgery restores competency to the lower esophageal sphincter (LES) with the intent of preventing retrograde flow of gastric contents into the esophagus. However, if there is excessive prevention of retrograde flow, patients lose the ability to properly vent during gastric distention and develop gas-bloat syndrome. In patients with normal gastric emptying (NGE), gas-bloat syndrome is somewhat ameliorated by enhanced gastric emptying [5]. However, the concern in patients with poor gastric motility and pyloric dysfunction is that the addition of the inability to vent will effectively trap gastric contents, exacerbating symptoms of DGE and leading to poor ARS outcomes.

Magnetic sphincter augmentation (MSA) is a safe and effective ARS with comparable outcomes to traditional antireflux surgeries [6]. Studies have demonstrated lower rates of postoperative gas-bloat syndrome after MSA compared to Nissen fundoplication [7, 8]. This is likely due to the magnetic properties of the MSA that allows for partial opening during gastric distention, preserving the ability to belch [9]. Therefore, it has been suggested that the MSA has a comparatively modest negative impact on gastric physiology and might be a more feasible choice in patients with gastroparesis. However, the relationship between DGE and MSA outcomes has not been previously studied. Therefore, we designed this study to assess the impact of objective DGE on outcomes after MSA compared to patients with NGE on GES.

## Methods

### Study population

This was a retrospective review of prospectively collected data of 775 patients who underwent MSA at Allegheny Health Network hospitals (Pittsburgh, PA) between 2014 and 2021. Patients with GERD who were 18 years or older with no history of prior foregut surgery who completed preoperative GES and had at least 1 year follow-up were included in this study. Patients with a history of esophageal or gastric surgical procedure; significant esophageal dysmotility and gross anatomic abnormality, such as esophageal stricture were not included in this study. This study was evaluated and approved by the Allegheny Health Network IRB (IRB Number 2020-064).

### Symptomatic and quality of life assessment

All patients were asked to complete Gastroesophageal Reflux Disease-Health-Related Quality of Life (GERD-HRQL) questionnaire preoperatively, at 6, 12 months and annually after surgery. The GERD-HRQL consists of 16 questions that specifically address GERD symptoms. Each question has a score ranging from 0 to 5. The best possible aggregate score is 0 (asymptomatic) and the worst score is 80 (very severe symptoms). A total score ≥ 15 is considered abnormal. Within the GERD-HRQL are specific questions pertaining to the symptoms of heartburn, regurgitation, dysphagia and gas-bloat. A score ≥ 4 of any of these symptom-specific items was considered a clinically significant symptom.

### Preoperative objective assessment

All patients underwent a comprehensive clinical evaluation with a focus on their foregut symptoms and antisecretory medication use. Patients also underwent routine preoperative objective assessment including the following tests:

- Videoesophagram was done to evaluate gross pharyngeal and esophageal motility, delineate anatomy, assess for any masses or mucosal lesions, diverticulum, and to evaluate hiatal hernia (HH) and esophageal stricture.
- Esophagogastroduodenoscopy with biopsy was used to assess the presence of esophagitis, Barrett’s esophagus, and the presence and size of an HH. HH size was recorded in centimeters based on the distance from the gastroesophageal junction (GEJ) to the crural impression.
- High-resolution manometry was used assess the esophageal body peristalsis (organization and pressure), as well as LES competency. Patients with severe esophageal body motility dysfunction were preferentially offered alternatives to MSA.
- Esophageal pH-monitoring was performed selectively using either Bravo pH monitoring (Medtronic, Minneapolis, MN) or pH impedance (Diversatek, Milwaukee, WI). Proton pump inhibitors (PPIs) were discontinued for 10 days before pH testing. Abnormal distal esophageal acid exposure was defined as a DeMeester score > 14.7.

### Gastric emptying scintigraphy (GES) technique and interpretation

Patients with non-specific symptoms common in GERD, but potentially suggestive of gastroparesis (e.g. nausea, vomiting, bloating) underwent gastric emptying scintigraphy. Patients ingested a standardized meal containing 1 mCi of technetium-99 m sulfur colloid. A series of anterior and posterior images were taken over the abdomen for 60 s immediately following ingestion, and then at hourly intervals for 4 h. The region containing the stomach was identified, and radiometric counts from this region immediately after ingestion were compared to the attenuation corrected counts at the hourly intervals to determine percent meal retention. A percent retention at 4 h ≤ 10% was considered normal gastric emptying (NGE), > 10% was considered delayed gastric emptying (DGE). In a subset of patients where only the report of the ‘time-to-50%-emptying’ (T_1/2_) was available, a T_1/2_ < 90 min was considered normal and T_1/2_ ≥ 90 min was delayed. Patients with preoperative DGE were compared to patients with preoperative NGE for analysis. Severely delayed gastric emptying was defined as percent retention at 4 h > 35%. Rapid gastric emptying was defined as a percent retention at 1 h < 60% and a normal 4-h percent retention.

### Device and surgical procedure

The LINX device (Ethicon, Johnson & Johnson; Shoreview, MN) is an expandable bracelet of titanium beads with magnetic cores that is placed around the esophagus at the esophagogastric junction (EGJ). The MSA is manufactured in different sizes, ranging from 13 to 17 beads. A manufacturer provided sizing device that measures the circumference in number of beads is used to assist in the selection of the appropriately sized device.

The implant procedure is performed laparoscopically and consists of complete mediastinal dissection, restoration of 3 cm of intraabdominal esophageal length, crural closure with interrupted posterior sutures and mobilization of the posterior vagus nerve.

The device placement is at or just above the level of the GEJ with the posterior vagus nerve trunk located on the outside of the magnetic ring.

### Postoperative outcomes assessment

Subjective postoperative outcomes were evaluated at routine visits at 2, 6 weeks, 6 months, and annually thereafter. Patients completed GERD-HRQL questionnaires and were assessed for resolution of their reflux symptoms, including heartburn, regurgitation, dysphagia, and gas-bloat; use of antisecretory medications; and procedure-related complications. Length of hospital stay, need for readmission within 90 days after operation, and need for postoperative dilation and device removal were also recorded. At 1 year after MSA, patients were approached for objective foregut evaluation using the same tests used in the preoperative evaluation.

### Statistical analysis

Baseline demographics and outcomes at 6 months and 1-year were compared between DGE and NGE patients. A subset of patients with 2-year follow-up were compared separately. Similar comparative analyses were performed between patients with severely delayed and delayed gastric emptying, and rapid and normal gastric emptying. Finally, a correlation analysis was performed between 4-h percent retention and pH-monitoring and GERD-HRQL data. Values for continuous variables are expressed as either mean (SD) or median with interquartile range when appropriate. Values for categorical variables are presented as frequency and percentage. Statistical analysis was performed by means of nonparametric tests, including Wilcoxon signed rank test and Fisher’s exact test when appropriate. A *p*-value < 0.05 was considered statistically significant. All statistical analyses were performed using SAS software (version 9.4, SAS Institute Inc., Cary, NC).

## Results

There were 131 patients (74.7% female) who underwent gastric emptying scintigraphy (GES) prior to MSA and completed at least 1-year follow-up. The median (IQR) age was 55.7 (44.9–62.9) and BMI was 29.1 (26.8–33.1). At a mean (SD) follow-up of 12.4 (1.7) months, median (IQR) GERD-HRQL total scores improved from 40 (26–55) to 8.5 (3–23), p < 0.0001. Freedom from PPI was achieved by 90.4% of patients with 80.2% patient satisfaction and 76.2% pH-normalization.

There were 26 (19.8%) patients with delayed gastric emptying (DGE) and 105 patients with NGE on GES. Baseline demographic and clinical characteristics of these two groups are compared in Table 1. Demographic and preoperative clinical findings were comparable between groups. Esophagitis was more common in patients with DGE (53.8%) than NGE (36.2%), but this did not reach significance (*p* = 0.119).

**Table 1:** Baseline demographic and clinical characteristics

	DGE (*n* = 26)	NGE (*n* = 105)	*p*-value
Age	53.2 (47–61)	56.4 (42–64)	1.000
Sex (Female)	21 (80.8%)	75 (71.4%)	0.459
BMI	32.0 (28–34)	28.9 (26–32)	0.088
BMI ≥ 30	15 (57.7%)	46 (43.8%)	0.273
Hiatal hernia	25 (92.3%)	96 (91.4%)	1.000
Hernia > 3 cm	3 (11.5%)	20 (19.0%)	0.565
Barrett’s	5 (18.5%)	21 (20.0%)	1.000
Esophagitis	15 (53.8%)	38 (36.2%)	0.119
Grade C/D	3 (11.5%)	8 (6.7%)	0.415
DeMeester score	25.4 (19–39)	31.1 (18–42)	0.646
GERD-HRQL total score	42.0 (24–59)	40.0 (26–55)	0.888

*DGE* delayed gastric emptying, *NGE* normal gastric emptying

The median (IQR) percent retention for 1, 2 and 4 h were significantly higher for DGE group **(**Table 2**)**. Patients with DGE were more likely to report symptoms of nausea and vomiting and they had a higher likelihood of complaining of more than one gastroparesis symptom **(**Table 2**)**.

**Table 2:** Baseline gastroparesis symptom and emptying data

	DGE (*n* = 26)	NGE (*n* = 105)	*p*-value
Gastric emptying scintigraphy			
1-h retention	81 (76–85)	66.9 (51–80)	0.0007
2-h retention	63 (49–74)	31 (16–45)	< 0.0001
4-h retention	24 (19–34)	1.0 (0–3)	< 0.0001
Gastroparesis symptoms			
Nausea	17 (65.4%)	31(29.5%)	0.0012
Vomiting	9 (34.6%)	15 (14.2%)	0.025
Bloating	22 (84.6%)	96 (91.4%)	0.288
≥ 1 Symptom	26 (100%)	105 (100%)	1.000
≥ 2 Symptoms	16 (61.5%)	26 (24.8%)	0.0007
3 Symptoms	6 (23.1%)	6 (5.7%)	0.014

*DGE* delayed gastric emptying, *NGE* normal gastric emptying

During the initial 90 days after surgery 5 (18.5%) patients with DGE and 3 (2.9%) patients with NGE required readmission to the hospital (*p* = 0.009). The indications for readmissions in DGE group were severe dysphagia, complicated by poor oral intake and dehydration, all successfully managed with fluid resuscitation and endoscopic dilation (*n* = 4) and surgical site infection (*n* = 1). The indications for readmissions in the NGE group were nausea and vomiting with dehydration, managed conservatively (*n* = 1), pneumonia (*n* = 1) and severe esophageal spasm, which was managed with endoscopic dilation (*n* = 1).

### MSA outcomes at 6 months, 1-year, and 2-years after surgery

Outcomes at 6 months after MSA are compared between groups in Table 3. The median GERD-HRQL total score was significantly higher in patients with DGE (*p* = 0.0013). Additionally, the heartburn score (0.0010) and gas-bloat score (0.033) were significantly higher in patient with DGE. Patients with DGE also had a higher need for dilation (*p* = 0.023). All other outcomes were similar between groups.

**Table 3:** 6-month outcomes after MSA

	DGE (*n* = 26)	NGE (*n* = 105)	*p*-value
GERD-HRQL total score	17.0 (10–29)	5.5 (3–16)	0.0013
GERD-HRQL heartburn item	1 (1–3)	0 (0–1)	0.0010
GERD-HRQL regurgitation item	1 (0–2)	0 (0–1)	0.039
GERD-HRQL dysphagia item	1.5 (1–3)	0 (0–2)	0.119
GERD-HRQL gas-bloat item	4 (2–5)	2 (1–3)	0.033
Freedom from PPI	76.9%	88.6%	0.397
Satisfaction	69.2%	87.6%	0.119
Need for dilation	40.7%	19.0%	0.023
Device removal	0%	1.0%	1.000

*DGE* delayed gastric emptying, *NGE* normal gastric emptying

Outcomes at 1-year are compared in Table 4. All outcomes were comparable between groups at 1-year. The rate of normalization of distal esophageal acid exposure was similar.

**Table 4:** 1-year outcomes after MSA

	DGE (*n* = 26)	NGE (*n* = 105)	*p*-value
GERD-HRQL total score	12.5 (5–27)	8.0 (3–18)	0.157
GERD-HRQL heartburn item	1 (0–2)	0 (0–2)	0.241
GERD-HRQL regurgitation item	1 (0–2)	0 (0–2)	0.672
GERD-HRQL dysphagia item	1.5 (0–2)	1 (0–2)	0.246
GERD-HRQL gas-bloat item	3 (1–3)	2 (1–3)	0.687
Freedom from PPI	84.0%	92.0%	0.257
Satisfaction	80.8%	79.8%	1.000
Device removal	7.4%	1.9%	0.176

*DGE* delayed gastric emptying, *NGE* normal gastric emptying

A subset of 68 (13 with DGE) patients were evaluated at two-years after MSA. Outcomes from this group are shown in Table 5. All outcomes were comparable between groups at 2-years.

**Table 5:** 2 year outcomes after MSA

	DGE (*n* = 13)	NGE (*n* = 55)	*p*-value
GERD-HRQL total score	14.5 (7–20)	9.0 (4–17)	0.187
GERD-HRQL heartburn item	1.5 (0–2)	1 (0–2)	0.221
GERD-HRQL regurgitation item	0 (0–2)	0 (0–1)	0.817
GERD-HRQL dysphagia item	1.5 (0–3)	1 (0–2)	0.638
GERD-HRQL gas-bloat item	2 (1–3)	2 (1–3)	0.980
Freedom from PPI	88.9%	78.9%	0.667
Satisfaction	90.9%	87.0%	1.000
Device removal	11.1%	3.8%	0.140

*DGE* delayed gastric emptying, *NGE* normal gastric emptying

**Fig. 1:**
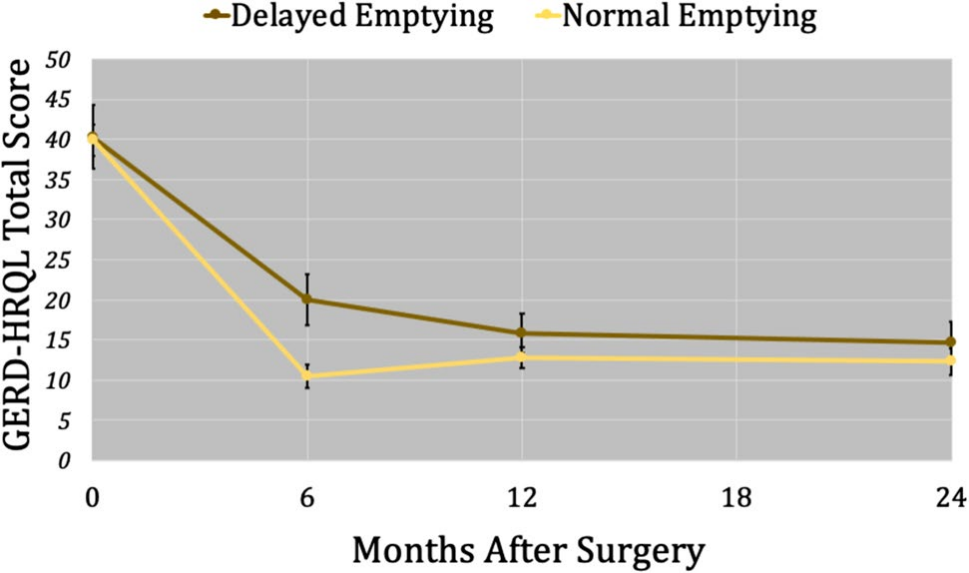
Mean GERD-HRQL total scores for normal and delayed gastric emptying groups at 6-month, 1-year and 2-year follow-up are shown with standard error bars. Delayed emptying was associated with a lower 6-month total score (*p* = 0.0013)

**Fig. 2:**
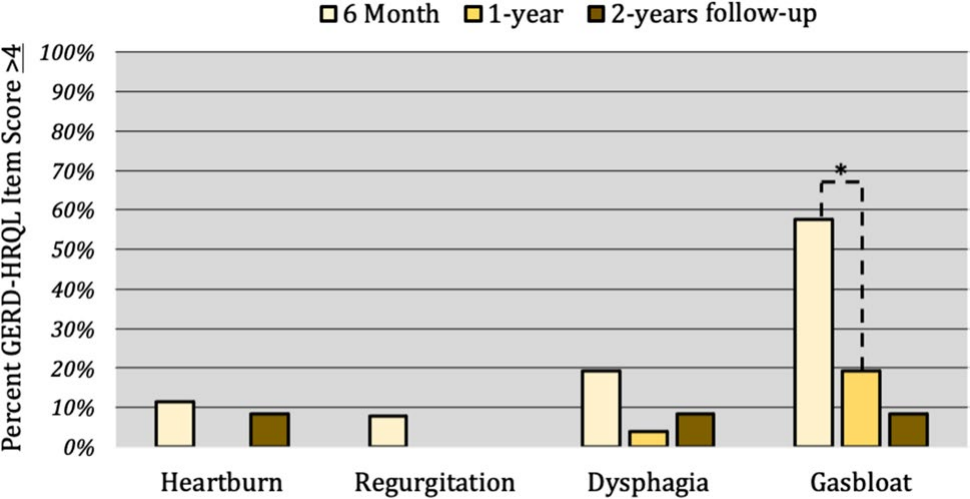
GERD-HRQL symptom-specific item scores ≥ 4 at 6-month, 1-year and 2-year follow-up are shown for patients who had delayed gastric emptying. The percent of patients who scored the gas-bloat item ≥ 4 significantly decreased between 6 months and 1 year (*p* = 0.004). No other changes in symptoms reached significance (*p* > 0.05)

Mean (SE) GERD-HRQL total scores at all time points are shown in Fig. 1. The prevalence of symptom-specific item scores rated ≥ 4 on the GERD-HRQL at all 3 outcomes time points are compared in Fig. 2. Among patients with delayed gastric emptying, the decrease in bloating from 6-month to 1-year was significant (*p* = 0.041). However, the differences in GERD-HRQL total score (*p* = 0.250) and heartburn score were not significant (*p* = 0.190). There was no difference between 1-year and 2-year GERD-HRQL total or symptom-specific item scores (*p* > 0.05).

### Severely delayed gastric emptying

There were only 4 patients with a 4-h retention > 35%. These patients with severely delayed gastric emptying had a higher GERD-HRQL total scores at 6 months and at 1-year compared to those with delayed but not severely delayed emptying **(**Table 6**)**. But these differences did not reach statistical significance. Freedom from PPI was significantly lower at 6 months (75 vs 87%, *p* = 0.014) and at 1-year (50 vs 92%, *p* = 0.046). There was a nonsignificant trend towards lower rates of normalization of distal esophageal acid exposure (33 vs 76.9%, *p* = 0.147) and higher rate of device removal (50 vs 14.8%, *p* = 0.119) in patients with severely DGE. No patients with severely delayed gastric emptying completed a 2-year follow-up.

**Table 6:** Comparison of outcomes between severe and not severe delayed emptying

	Severely delayed (*n* = 4) (4-h retention > 35%)	Not severely delayed (*n* = 22) (10% < 4-h retention < 35%)	*p*-value
6-month outcomes			
GERD-HRQL total score	24 (13–34)	8.0 (3–18)	0.084
Freedom from PPI	75.0%	86.5%	0.014
Satisfaction	50.0%	84.1%	0.143
1-year outcomes			
GERD-HRQL total score	22 (15–25)	8 (3–24)	0.194
Freedom from PPI	50.0%	91.9%	0.046
Satisfaction	50.0%	79.8%	0.197
Device removal	50.0%	14.8%	0.119

### Rapid gastric emptying

There were only 2 patients who met criteria for rapid gastric emptying. One patient’s GERD-HRQL total score decreased from 70 preoperatively to 34 at 6 months, 26 at 1-year and 10 by 2-years. They reported satisfaction with their postoperative outcomes and remained off PPIs. The other patient had a preoperative score of 43, which decreased to 15 by 6 months, but increased to 45 by 1-year and increased to 68 by 2-years. They remained dissatisfied throughout their postoperative follow times and resumed PPIs. This patient has undergone multiple dilations and reports some benefit.

### Correlation between 4-h percent retention and outcome

**Fig. 3:**
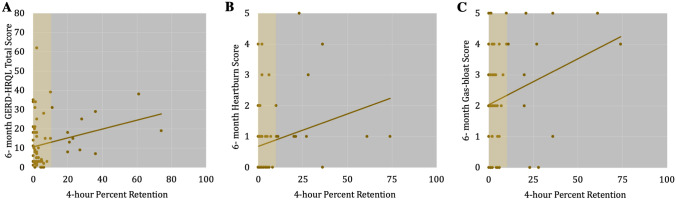
**A** Correlation between GERD-HRQL total score at 6 months and preoperative 4-h percent retention on gastric emptying scintigraphy (*R* = 0.253, *p* = 0.039). Correlation between **(B)** GERD-HRQL Heartburn item score at 6 months (*R* = 0.242, *p* = 0.048) and **C** GERD-HRQL Gas-bloat item score at 6 months (*R* = 0.246, *p* = 0.045) with preoperative 4 h percent retention on gastric emptying scintigraphy

There were significant, but weak correlations between 4-h percent retention and 6-month GERD-HRQL total score (*R* = 0.253, 95% CI (0.09–0.41), *p* = 0.039), heartburn-item (*R* = 0.242, 95% CI (0.07–0.40), *p* = 0.048) and gas-bloat item (*R* = 0.246, 95% CI (0.08–0.40), *p* = 0.045) **(**Fig. 3**)**. Total scores and 4-h percent retention were not correlated at 1-year (*p* = 0.557) or 2-years (*p* = 0.517). There were no significant correlations between 4-h percent retention and postoperative DeMeester score (*p* = 0.514) or percent total time with pH < 4 (*p* = 0.880).

## Discussion

The relationship between delayed gastric emptying (DGE) and antireflux surgery (ARS) outcome is a topic steeped in controversy. Conceptually, concern for the integrity of a repair in a patient with chronic vomiting and retching is warranted, as these events cause vigorous hiatal motion and have been associated fundoplication disruption [10, 11]. In addition, there is concern that symptoms due to persistent delayed gastric emptying may diminish patient perceived efficacy of the surgery, leading to an appraisal of failure. Previous studies have focused on the relationship between DGE and fundoplication. The magnetic sphincter augmentation (MSA) is the most recent widely accepted antireflux surgery (ARS) added to the foregut surgeon’s armamentarium. Despite more than 10-years clinical experience with this procedure, there is limited data on the impact of delayed gastric emptying on the outcome of MSA. The current study was designed to address this knowledge gap. Our findings suggest that DGE does have a negative effect on MSA outcomes, early on. Patients with DGE had a higher rate of 90-days readmissions. They also experienced a higher rate of gas-bloat in the first 6 months, but this symptom typically resolves over time without further intervention. As a result, outcomes were comparable by 1-year and remained durable at 2-years.

We found that overall disease-specific quality of life scores at 6 months were significantly higher in patients with DGE, with the greatest differences seen in bloating and heartburn symptoms. This was despite comparable preoperative scores for these two components. Diminished outcomes after MSA in patients with DGE is a novel finding in the literature. However, this result is consistent with previous studies of the relationship between DGE and fundoplication. One such study examined 141 patients with gastroparesis symptoms who underwent fundoplication with or without pyloroplasty. The authors compared patients with and without objective DGE and found that patients with objective evidence of DGE who underwent fundoplication alone had the worst reflux symptom control with 25% of these patients demonstrating symptomatic failure. However, this difference was not significant between groups [11]. Another study of gastric emptying in 16 patients with persistent symptoms after fundoplication compared to 21 symptom free post-fundoplication patients found that persistent symptoms were associated with significantly longer gastric emptying times, suggesting a relationship between emptying time and failure [2]. Another study of pre- and postoperative gastric emptying found that all patients whose preoperative delayed gastric emptying failed to normalize postoperatively had persistent symptoms after ARS. These findings suggest that persistent delayed gastric emptying symptoms may diminish ARS outcome. Although studies have demonstrated that emptying accelerates following fundoplication, relying upon this effect alone may result in severe gas-bloat in patient whose postoperative emptying remains insufficient [5]. This concern was validated by Maddern et al. who demonstrated that failure to control symptomatic and objective reflux after fundoplication was associated with longer postoperative gastric emptying times [2]. Based on these findings some surgeons have recommended performing a pyloric drainage procedure at the time of ARS, while others have recommended a more tailored approach, reserving pyloroplasty for severe DGE [1, 11, 12]. Due to MSA being an implanted device that is subject to risk of infection, pyloroplasty is better performed during a separate surgery, if deemed necessary. Based on the findings of the present study, patients with delayed gastric emptying may have diminished outcomes early on in their postoperative course.

Overtime patients with DGE improved from a symptomatic standpoint, reaching comparable outcomes at 1 and 2-years. Bloating is a common symptom after antireflux surgery and was one of the significant symptoms at 6 months. However, numerous studies have demonstrated that postoperative bloating and in particular the gas-bloat syndrome is worst initially after surgery and largely resolves over time. Although previous studies have demonstrated lower rates of gas-bloat syndrome in patients with a magnetic sphincter augmentation, a recent study from our center demonstrated gas-bloat after MSA is associated with suboptimal outcomes [13]. Further, preoperative bloating, a common symptom of delayed gastric emptying, was associated with gas-bloat syndrome after MSA. In a patient with delayed gastric emptying, this additional bloating may promote reflux of gastric contents and result in the higher rates of heartburn that we observed. Following fundoplication, the development of an inability to belch may exacerbate these symptoms in a patient with delayed gastric emptying, resulting in a relationship between DGE and outcomes that persists overtime. By contrast, MSA preserves the ability to belch, alleviating some bloating. As this bloating resolves overtime, the increased gastric distention is alleviated, and symptomatic outcomes become equivalent.

A similar temporal decline in the impact of DGE on ARS outcomes has not been reported in the literature on fundoplication. Furthermore, some studies have demonstrated an association between DGE and diminished outcomes more than 1-year after surgery. This inconsistency is most likely due to the fundamental differences between fundoplication and MSA with regard to in their impact on gastric physiology. Construction of a fundoplication repurposes gastric tissue to augment the reflux barrier, and in doing so reduces the volume of the stomach, which is thought to be a mechanism of enhancing gastric emptying. One study measuring gastric emptying, volume and compliance before and after fundoplication found that postoperative decreases in gastric volume were associated with corresponding increases in gastric emptying [14]. By contrast, MSA, is an extraluminal expandable implant, that preserves gastric anatomy. Therefore, gastric volume reduction is not a factor in post-MSA gastric physiology, a fact that likely contributes to the differences in the fundoplication and the MSA relationship with DGE.

Debate over the relationship between gastric emptying and GERD is an offshoot of the debate over the etiology of GERD. The association between transient lower esophageal sphincter relaxations (TLESR) and GERD is clear. Some studies have suggested that reduced rates of TLESRs after fundoplication is one of the mechanisms by which ARS is effective; however, data has been inconsistent and manometry data have more clearly demonstrated a relationship between restoration of the sphincter and reflux control [15, 16]. Delayed gastric emptying is associated with increased TLESRs, leading to the hypothesis that DGE leads to worse GERD [17]. Studies have demonstrated that the fundoplication retains some of its neurohormonal ability to relax during events such as TLESRs [18]. Postprandial cholecystokinin stimulates the fundus to relax and causes TLESRs, a function which is preserved following fundoplication [19, 20]. Patients with DGE may have higher degree of postprandial gastric distension with more frequent TLESRs, resulting in recurrent reflux even after fundoplication. By contrast, MSA does not relax at any point being made of titanium beads. Rather during deglutition or gastric venting the beads begin to open, resulting in an exponential decrease in magnetic forces [9]. Therefore, MSA is not impacted by these factors, which is likely the reason why there was no lasting difference in MSA outcomes between those with and without DGE. Therefore, conventional wisdom suggests that MSA is a more appropriate antireflux surgery in patients with objective DGE.

Despite studies demonstrating an association between DGE and TLESRs, previous studies have not demonstrated a strong correlation between DGE and distal esophageal acid exposure. In fact, some studies have demonstrated frequent TLESRs in patients with enhanced gastric emptying while others have suggested that retained food may act as a buffer, reducing the pH of potential refluxate in patients with delayed gastric emptying [21]. Consistent with these findings, we found no significant relationship between 4-h percent gastric retention and DeMeester score or percent total time with pH < 4, suggesting that objective reflux control and gastric emptying are independent factors.

This study is not without its limitations including its retrospective nature and small sample size. There were only 4 patients with severely delayed gastric emptying and compared to their non-severely delayed counterparts only freedom from PPI was significantly diminished. However, there was a non-significant trend towards worse symptomatic outcomes at 6 months and 1-year and a higher removal rate. Previous studies have suggested that severely delayed emptying has a more profound impact on fundoplication outcomes [2]. The present study found a similar trend with MSA but was likely too underpowered to fully demonstrate the relationship between severely delayed emptying and MSA outcomes. Another limitation was the lack of a randomized or universal GES protocol. GES at our institution is ordered at the discretion of the surgeon, in response to symptoms that may be concerning for an underlying gastroparesis. However, these symptoms are non-specific and very common in patients with reflux, affecting up to 40% of patients with GERD [22]. This is the reason we chose to focus on objective delayed emptying and explains the high rate of NGE in this symptomatic population. However, replication of our results in a population who underwent GES regardless of any other factors is warranted to control for possible selection bias.

Rapid gastric emptying is a poorly understood clinical entity that previous studies have linked to more frequent upright reflux [23]. Some studies have demonstrated a strong association between rapid emptying and fundoplication, and other studies have suggested an association between emptying time and ARS outcomes [2, 24]. However, in this study we only identified two patients with rapid gastric emptying with mixed outcomes. Further research is necessary to elucidate the clinical relevance of rapid gastric emptying and its impact on ARS outcome.

## Conclusion

There remains debate as to whether DGE may increase the risk of postoperative complications such as gas-bloat and lead to suboptimal ARS outcomes. However, this study demonstrated that magnetic sphincter augmentation is an effective treatment for those with concomitant GERD and DGE. Patients with DGE experienced higher rates of 90-day readmission, and at 6-month follow-up reported more heartburn and gas-bloat with diminished symptomatic improvement. However, by 1-year outcomes improved and were comparable, and these results were durable at 2-years. There was a non-significant trend for worse outcomes at 6 months and 1 year, with higher device removal rates among patients with severely delayed emptying. Therefore, MSA is a viable option for patients with mild-to-moderate DGE. However, it is important to manage expectations, and inform patients that the full benefits of MSA may not be realized until 1 year after surgery. Further research is necessary to elucidate the impact of severely delayed emptying on outcome; however, data from this study suggests that outcomes may be diminished. Therefore, a more cautious approach is warranted in patients with severe gastroparesis.

## References

[1] Masqusi S, Velanovich V (2007). Pyloroplasty with fundoplication in the treatment of combined gastroesophageal reflux disease and bloating. World J Surg.

[2] Maddern G, Jamieson G, Chatterton B, Collins P (1985). Is there an association between failed antireflux procedures and delayed gastric emptying?. Ann Surg.

[3] Alexander F, Wyllie R, Jirousek K, Secic M, Porvasnik S (1997). Delayed gastric emptying affects outcome of nissen fundoplication in neurologically impaired children. Surgery.

[4] Brown RA, Wynchank S, Rode H, Millar AJ, Mann MD (1997). Is a gastric drainage procedure necessary at the time of antireflux surgery?. J Pediatr Gastroenterol Nutr.

[5] Bais JE, Samsom M, Boudesteijn EA, van Rijk PP, Akkermans LM, Gooszen HG (2001). Impact of delayed gastric emptying on the outcome of antireflux surgery. Ann Surg.

[6] Lipham J, Taiganides P, Louie B, Ganz R, DeMeester T (2015). Safety analysis of first 1000 patients treated with magnetic sphincter augmentation for gastroesophageal reflux disease. Dis Esophagus.

[7] Warren HF, Reynolds JL, Lipham JC, Zehetner J, Bildzukewicz NA, Taiganides PA, Mickley J, Aye RW, Farivar AS, Louie BE (2016). Multi-institutional outcomes using magnetic sphincter augmentation versus nissen fundoplication for chronic gastroesophageal reflux disease. Surg Endosc.

[8] Reynolds JL, Zehetner J, Wu P, Shah S, Bildzukewicz N, Lipham JC (2015). Laparoscopic magnetic sphincter augmentation vs laparoscopic Nissen fundoplication: a matched-pair analysis of 100 patients. J Am Coll Surg.

[9] Eriksson SE, Jobe BA, Ayazi S (2022). Magnetic sphincter augmentation and high-resolution manometry: impact of biomechanical properties on esophageal motility and clinical significance for selection and outcomes. Dis Esophagus.

[10] Richards CA (2019). Does retching matter? Reviewing the evidence—physiology and forces. J Pediatr Surg.

[11] Khajanchee YS, Dunst CM, Swanstrom LL (2009). Outcomes of Nissen fundoplication in patients with gastroesophageal reflux disease and delayed gastric emptying. Arch Surg.

[12] Swanstrom LL (2001). Management of patients with gastroesophageal reflux disease and esophageal or gastric dysmotility. J Gastrointest Surg.

[13] Eriksson S, Ayazi S, Byrne E, Maurer N, Abu-Nuwar MR, Zheng P, Jobe B (2022). Gas-bloat syndrome after magnetic sphincter augmentation: incidence, risk factors, and impact on surgical outcomes. J Am Coll Surg.

[14] Bustorff-Silva J, Perez CA, Fonkalsrud EW, Hoh C, Raybould HE (1999). Gastric emptying after fundoplication is dependent on changes in gastric volume and compliance. J Pediatr Surg.

[15] Kessing BF, Bredenoord AJ, Schijven MP, van der Peet DL, van Berge Henegouwen MI, Smout AJ (2015). Long-term effects of anti-reflux surgery on the physiology of the esophagogastric junction. Surg Endosc.

[16] Bonavina L, Evander A, DeMeester TR, Walther B, Cheng S-C, Palazzo L, Concannon JL (1986). Length of the distal esophageal sphincter and competency of the cardia. Am J Surg.

[17] Holloway RH, Hongo M, Berger K, McCallum RW (1985). Gastric distention: a mechanism for postprandial gastroesophageal reflux. Gastroenterology.

[18] Jiang Y, Sandler B, Bhargava V, Mittal RK (2011). Antireflux action of nissen fundoplication and stretch-sensitive mechanism of lower esophageal sphincter relaxation. Gastroenterology.

[19] Scheffer R, Akkermans L, Bais J, Roelofs J, Smout A, Gooszen H (2002). Elicitation of transient lower oesophageal sphincter relaxations in response to gastric distension and meal ingestion. Neurogastroenterol Motil.

[20] Boeckxstaens G, Hirsch D, Fakhry N, Holloway R, D’Amato M, Tytgat G (1998). Involvement of cholecystokininA receptors in transient lower esophageal sphincter relaxations triggered by gastric distension. Am J Gastroenterol.

[21] Emerenziani S, Sifrim D (2005). Gastroesophageal reflux and gastric emptying, revisited. Curr Gastroenterol Rep.

[22] Mccallum RW, Berkowitz DM, Lerner E (1981). Gastric emptying in patients with gastroesophageal reflux. Gastroenterology.

[23] Little AG, DeMeester TR, Kirchner PT, O'Sullivan GC, Skinner DB (1980). Pathogenesis of esophagitis in patients with gastroesophageal reflux. Surgery.

[24] Gomez Cifuentes J, Radetic M, Lopez R, Gabbard S (2019). Clinical predictors of rapid gastric emptying in patients presenting with dyspeptic symptoms. Dig Dis Sci.

